# Detecting AGG Interruptions in Females With a FMR1 Premutation by Long-Read Single-Molecule Sequencing: A 1 Year Clinical Experience

**DOI:** 10.3389/fgene.2018.00150

**Published:** 2018-05-16

**Authors:** Simon Ardui, Valerie Race, Thomy de Ravel, Hilde Van Esch, Koenraad Devriendt, Gert Matthijs, Joris R. Vermeesch

**Affiliations:** Center for Human Genetics, University Hospitals Leuven, University of Leuven, Leuven, Belgium

**Keywords:** AGG interruptions, fragile X syndrome, single-molecule real-time sequencing, fragile X premutation, genetic counseling, *FMR1*, CGG repeat

## Abstract

The fragile X syndrome arises from the *FMR1* CGG expansion of a premutation (55–200 repeats) to a full mutation allele (>200 repeats) and is the most frequent cause of inherited X-linked intellectual disability. The risk for a premutation to expand to a full mutation allele depends on the repeat length and AGG triplets interrupting this repeat. In genetic counseling it is important to have information on both these parameters to provide an accurate risk estimate to women carrying a premutation allele and weighing up having children. For example, in case of a small risk a woman might opt for a natural pregnancy followed up by prenatal diagnosis while she might choose for preimplantation genetic diagnosis (PGD) if the risk is high. Unfortunately, the detection of AGG interruptions was previously hampered by technical difficulties complicating their use in diagnostics. Therefore we recently developed, validated and implemented a new methodology which uses long-read single-molecule sequencing to identify AGG interruptions in females with a *FMR1* premutation. Here we report on the assets of AGG interruption detection by sequencing and the impact of implementing the assay on genetic counseling.

## Introduction

Variability of the CGG tandem repeat in the 5′ untranslated region (UTR) of the fragile X mental retardation gene (*FMR1*; MIM# 309550) is associated with various disorders. Whereas most individuals in the general population have around 30 CGG repeats (<45 repeats), patients with fragile X syndrome (FXS; MIM# 300624) carry large, full expansions sized above 200 repeats (Oberlé et al., 1991; Verkerk et al., 1991). These large expansions are usually epigenetically silenced thereby inhibiting the production of fragile X mental retardation protein (FMRP) (Pieretti et al., 1991). The absence of protein evokes FXS, a neurodevelopmental disorder characterized by intellectual disability, emotional problems, autism, hyperactivity, hypersensitivity and mild dysmorphic features (Penagarikano et al., 2007). Premutation carriers represent yet another group with repeat sizes varying between 55 and 200 repeats, and might be affected by fragile X-associated tremor/ataxia syndrome (FXTAS; MIM# 300623) or fragile X-associated primary ovarian insufficiency (FXPOI; MIM# 311360) amongst other medical problems (Sullivan et al., 2005; Van Esch, 2006; Hagerman and Hagerman, 2015). In between the premutation alleles (55–200 repeats) and normal alleles (<45 repeats) an intermediate zone (45–54 repeats) exists. Although carriers of intermediate alleles are generally believed to be healthy, some reports have shown that these alleles might be associated with parkinsonism (Loesch et al., 2009) and FXTAS, although with a milder phenotype, less frequently and at later age-of-onset (Hall et al., 2012; Liu et al., 2013). Hence, depending on the size of the *FMR1* CGG repeat, an individual will be affected by different disorders varying both mechanistically and phenotypically.

The *FMR1* CGG repeat is susceptible to meiotic instability which is reflected by repeat size differences between parents and offspring. Normal and intermediate alleles (<55 repeats) are usually inherited stably or differ at most a few repeats (Nolin et al., 2013). In both male and female premutation carriers repeat expansions and contractions are common (Nolin et al., 2003). Furthermore, women carrying a premutation allele are at risk of transmitting a full mutation to their offspring. With a reported frequency of around 1 in 200 females carrying a premutation, a significant fraction of the female population is at risk of having children with FXS (Cronister et al., 2008; Tassone et al., 2012). This risk depends on the repeat size and the number of AGG triplets interrupting the repeat whereby larger repeats with fewer AGGs have the highest expansion risks (Yrigollen et al., 2014a; Nolin et al., 2015). These AGGs intersperse the CGG repeat every 9 or 10 CGG repeats at the 5' end where their presence reduces repeat instability (Eichler et al., 1994; Yrigollen et al., 2014a; Nolin et al., 2015). The influence of AGG interruptions is the most profound for alleles ranging from 60 to 85 repeats. For instance, the risk of transmitting a full mutation for a woman with 75 repeats and two AGG triplets is 12%, but this increases to 77% if no AGG interruptions are present (Yrigollen et al., 2012). Some studies have reported that maternal age can also influence the expansion risk (Yrigollen et al., 2014a), but this could not be confirmed by others (Nolin et al., 2015). Hence, further large scale studies are needed to solve this issue. For small (<60 repeats) or large (>85 repeats) premutation alleles the influence of AGG interruptions on the expansion risk is only minor. Alleles smaller than 60 repeats have only an expansion risk of 2.6%, even in the absence of AGG repeats while large alleles on the contrary have an expansion risk higher than 60%, even when two stabilizing AGGs are present (Yrigollen et al., 2012).

In genetic counseling it is important to provide a female premutation carrier with an accurate estimate of her expansion risk because this influences her reproductive planning. When the expansion risk is high, women might opt for preimplantation genetic diagnosis (PGD) where one could select for unaffected males or non-carrier female embryos (Sermon et al., 1999; Burlet et al., 2006). Although the detection of large CGG alleles could be very challenging in a single cell picked from an early embryo, this can now be circumvented by making use of new haplotyping methods (Natesan et al., 2014; Zamani Esteki et al., 2015; Dimitriadou et al., 2017). If the risk of having a child with FXS is low, a women could choose for normal conception, optionally combined with invasive prenatal diagnosis to screen the fragile X status of their fetus (Biancalana et al., 2015). Therefore, it is important that genetic laboratories use both repeat size and the number of AGGs to assess the expansion risk. The repeat size can easily be determined by PCR-based methods and/or Southern blot, but the detection of AGG interruptions is technically challenging. This has hampered the clinical uptake of this information. Interruption analysis can be done by first- and second –generation triplet-primed PCR methods, but unfortunately these only provide an indirect indication of the presence of AGG interruptions (Chen et al., 2010; Hayward and Usdin, 2017). Interpreting the results of these assays is complicated in females who carry two X-chromosomes each containing a different CGG repeat with its own set of AGG triplets. To overcome these problems, a novel methodology was developed based on long-read single-molecule sequencing by Pacific Biosciences. This technology generates long reads (>20 kb), is suited to analyze GC-rich regions (like the *FMR1* CGG repeat) and able to detect embedded AGG interruptions (Travers et al., 2010; Loomis et al., 2013; Ardui et al., 2017). The generated single-molecule reads are separated according to the X-chromosome from which they originate, whereafter the exact repeat structure can be determined for each allele individually. Long-read single-molecule sequencing is the only technology so far that can separate the two repeats derived from different X-chromosomes, and hence generates superior results compared to PCR-based assays (Ardui et al., 2017). Furthermore, long-read single-molecule sequencing is diagnostically applicable and different applications are presently being implemented in human genetic diagnostics (Ardui et al., 2018).

The undisputed impact of AGG triplets on the expansion risk estimates of female premutation carriers along with good AGG interruption detection by single-molecule sequencing prompted us to implement this assay for diagnostic use for all female carriers with an intermediate and a premutation allele (45–200 repeats). By doing so we improved the risk assessments for genetic counseling and positively impacted the management of the disorder. We summarize our experience with the use of AGG triplets in the clinic after 1 year during which we analyzed 51 patients.

## Materials and methods

### Patients

The 51 female patients were ascertained from January to December 2017 at the Center for Human Genetics, KU Leuven, UZ Leuven, Belgium. The patients were referred for diagnostic testing because of either diagnostic work-up in the fertility clinic, POI, intellectual disability or a family history of FXS. Fifty patients carried a normal allele and an intermediate (26) or premutation allele (24). Also 1 female carrying two premutations was included in this study. The study was approved by the local Ethics Committee and consent was obtained from the patients, both informed and written.

### DNA sampling

For all 51 females DNA was isolated from peripheral white blood cells according to standard procedures. From one pregnant female patient a chorionic villi sample (CVS) sample was received. Villi from CVS were separated from maternal tissue under a microscope to minimize maternal contamination. Two to four villi were provided for DNA extraction.

### Molecular analysis

The repeat size was determined by (TP-)PCR for all samples. The structure of the CGG repeat in the *FMR1* gene was then determined for all intermediate and premutation carriers by sequencing according to the previously published method (Ardui et al., 2017). In brief, the *FMR1* CGG repeat was amplified by PCR during which a barcode was incorporated. After pooling different amplicons together, long-read single-molecule sequencing was performed on a PacBio RSII system. This generates long reads which span each PCR molecule multiple times thereby generating a highly accurate consensus sequence. This technology is “single-molecule” which allows the unambiguous separation of the CGG repeats derived from the two different X-chromosomes. Hence, after sequencing, the complete repeat structure could be reconstructed for both X-chromosomes revealing the repeat size and the AGG interruption pattern. The sizes determined by PCR control runs and single-molecule sequencing matched perfectly.

### Genetic counseling

Females with a premutation were offered genetic counseling. If the patient was considering having children, an accurate assessment of the risk that their premutation would expand to a full mutation was provided based on both the *FMR1* CGG repeat size and the number of AGG interruptions.

## Results

AGG analysis was implemented diagnostically in order to more accurately assess the risk that offspring of premutation carriers will be affected by FXS and thus improve genetic counseling. We report the results of AGG interruption detection in 51 females with intermediate or premutation alleles. Long-read single-molecule sequencing was used to determine the AGG triplets as it allows the determination of the exact *FMR1* repeat structure for each individual allele.

The results of the *FMR1* CGG repeat analysis are summarized in Table 1. Fifty females carried a normal allele and an intermediate (26) or a premutation allele (24), while 1 female carried two premutation alleles. Therefore, the total number of premutation alleles is also 26 (Table 1). The normal alleles of all 50 females ranged between 20 and 40 repeats and are interspersed with 0, 1, 2 or 3 AGGs. Two different clusters were identified within the structures of the normal alleles: a smaller group (20–24 repeats) interrupted by 1 AGG (11 patients) and a larger group (30–34 repeats) interrupted by 2 AGGs (29 patients), in line with previously published results (Eichler et al., 1996; Chen et al., 2003). The remaining ten normal alleles are more distributed in size and number of AGGs (Table 1; Eichler et al., 1996). From the 26 intermediate alleles and 26 premutation alleles (from 25 females), the majority (45) were interrupted by 1 or 2 AGG interruptions.

**Table 1:** Overview of the repeat size and the number of interrupting AGG triplets for 51 females.

	**# AGG triplets**				
**Repeat range**	**0**	**1**	**2**	**3**	**# alleles**
**Normal**					**50**
20–24	1	11	0	0	12
25–29	1	0	3	0	4
30–34	0	2	29	0	31
35–39	0	0	2	0	2
40–44	0	0	0	1	1
**Gray-zone**					**26**
45–49	1	3	14	1	19
50–54	0	4	3	0	7
**Premutation**					**26**
55–59	1	3	6	0	10
**60–64**	**0**	**1**	**0**	**0**	**1**
**65–69**	**2**	**0**	**3**	**0**	**5**
**70–74**	**1**	**0**	**3**	**0**	**4**
**75–79**	**0**	**0**	**2**	**0**	**2**
**80–84**	**0**	**0**	**2**	**0**	**2**
85–89	0	0	1	0	1
90–94	0	0	0	0	0
95–100	1	0	0	0	1
**# alleles with N AGG triplets**	8	24	68	2	

*Fifty females carry a normal allele (<45 repeats) and an intermediate (45–54 repeats) or a premutation allele (55–200 repeats) and 1 female carries two premutation alleles. The repeat range where the number of AGG triplets has the largest impact on the expansion risk is depicted in bold*.

Information on AGG interruptions is vital for females carrying a premutation allele because of the risk of transmitting a full mutation to their children. For the 13 females with a premutation allele ranging between 60 and 84 repeats, knowing the AGG status was reassuring for these carrying 2 AGGs, while it alerted females without any AGG triplets. For example, female 1 (Table 2) presented with an allele of 69 CGG repeats without interruptions. This female opted for PGD because she has a relatively high expansion risk (22,9%) in combination with a reduced fertility. Female 2 (Table 2) carried two premutation alleles (65 and 73 repeats) and chose for PGD because she also carried a translocation. For this female, AGG interruption analysis was performed to determine which allele had the lowest expansion risk. As sequencing revealed that two AGGs were present in each allele, embryos carrying the smallest allele of 65 repeats with 2 AGG's could be prioritized for transfer during PGD. Female 3 (Table 2) carried a premutation allele of 69 repeats interrupted with 2 AGG triplets. This female only has a low expansion risk of 0.5% and hence decided to choose for a natural pregnancy. Invasive prenatal follow-up of the ensuing pregnancy showed that the fetus inherited the normal copy. Two females carried a repeat above 85 repeats and hence invoked a high expansion risks: 100% for the female with 95 repeats without AGGs and 60% for the female with 89 repeats and 2 AGGs (female 4, Table 2). Although the high expansion risk of female 4, she became pregnant naturally. Fortunately, invasive prenatal follow-up showed the premutation allele even contracted in the female fetus (female 5, Table 2), most likely to the allele containing 66 repeats and 2 AGG's. A contraction to the allele with 44 repeats and 1 AGG is less likely, but cannot be excluded because the DNA of the father was not available.

**Table 2:** Overview of the *FMR1* CGG repeat structure for patient 1–10.

	**Normal**			**Premutation**		
**Female**	**Repeat length**	**# AGGs**	**Repeat structure**	**Repeat length**	**# AGGs**	**Repeat structure**
1	22	1	(CGG)_9_(AGG)_1_(CGG)_12_	69	0	(CGG)_69_
2	65	2	(CGG)_9_(AGG)_1_(CGG)_9_(AGG)_1_ (CGG)_45_	73	2	(CGG)_9_(AGG)_1_(CGG)_7_(AGG)_1_ (CGG)_55_
3	33	2	(CGG)_9_(AGG)_1_(CGG)_9_(AGG)_1_ (CGG)_13_	69	2	(CGG)_9_(AGG)_1_(CGG)_9_(AGG)_1_ (CGG)_49_
4	30	2	(CGG)_10_(AGG)_1_(CGG)_9_(AGG)_1_ (CGG)_9_	89	2	(CGG)_9_(AGG)_1_(CGG)_9_(AGG)_1_ (CGG)_69_
5	44	1	(CGG)_9_(AGG)_1_(CGG)_34_	66	2	(CGG)_9_(AGG)_1_(CGG)_9_(AGG)_1_ (CGG)_46_
6	32	2	(CGG)_9_(AGG)_1_**(CGG)**_12_(AGG)_1_ (CGG)_9_	45	2	(CGG)_9_(AGG)_1_(CGG)_9_(AGG)_1_ (CGG)_25_
7	34	2	(CGG)_9_(AGG)_1_**(CGG)**_14_(AGG)_1_ (CGG)_9_	49	1	(CGG)_9_(AGG)_1_(CGG)_39_
8	30	2	(CGG)_10_(AGG)_1_(CGG)_9_(AGG)_1_ (CGG)_9_	49	3	(CGG)_10_(AGG)_1_(CGG)_9_(AGG)_1_ **(CGG)**_18_(AGG)_1_(CGG)_9_
9	30	1	**(CGG)**_20_(AGG)_1_(CGG)_9_	69	2	(CGG)_9_(AGG)_1_(CGG)_7_(AGG)_1_ (CGG)_51_
10	40	3	(CGG)_10_(AGG)_1_(CGG)_9_(AGG)_1_ (CGG)_9_(AGG)_1_(CGG)_9_	45	2	(CGG)_7_**CTG**(CGG)_1_(AGG)_1_ (CGG)_9_(AGG)_1_(CGG)_25_

Sequencing of *FMR1* CGG repeats not only identified AGG triplets, but any sequence variation at this locus. The *FMR1* structure is most commonly built up from (CGG)_9_AGG and (CGG)_10_AGG building blocks. This was confirmed in most of the females of our cohort. However, 4 females (female 6–9, Table 2) carried a less common AGG interruption pattern. Female 10 (Table 2) carries an intermediate allele with 45 repeats and two AGG triplets, but also harbored a CTG interruption (Table 2).

## Discussion

Technical limitations have hampered the diagnostic uptake of AGG analysis so far, but this is now overcome by a novel single-molecule sequencing approach (Ardui et al., 2017). Long-read single-molecule sequencing generates high quality results and permits to construct unambiguously the repeat structure for both X-chromosomes in females. Incorporating AGG analysis into *FMR1* diagnostic work-up allows accurate risk estimates for having a child with FXS which greatly improved genetic counseling for woman carrying a premutation (Yrigollen et al., 2014a; Biancalana et al., 2015; Nolin et al., 2015). Here, we report the results of AGG interruption analysis of the first 51 females with an intermediate or premutation allele which have been collected at the Center of Human Genetics, KU Leuven, UZ Leuven (Belgium) during 1 year.

The impact of AGG interruptions is the most profound for females carrying a premutation sized between 60 and 84 repeats within which 13 females of our cohort fitted. From these 13 females, 3 females carried pure CGG repeats and hence have relatively high expansion risks ranging from 23 to 50% (Yrigollen et al., 2012, 2014a). These females with a high risk of having a child with FXS might opt for PGD where one can select for non-affected male- or carrier female embryos. The other 10 females had either 1 but most often 2 AGG interruptions and hence have more moderate expansion risks, except the two females with 80–84 repeats and 2 AGGs who also have around 30% chance their allele will expand into a full mutation (Yrigollen et al., 2012, 2014a). We conclude that the more accurate risk estimates provided to the females with a premutation simplified choosing the most appropriate reproductive strategy.

Single-molecule sequencing provides a direct read-out of the *FMR1* CGG repeat and hence allows to grasp the complete repeat sequence. Most of our normal, intermediate and premutation alleles are constructed with CGG_9_AGG or CGG_10_AGG building blocks, concordant with previously published reports (Eichler et al., 1996; Yrigollen et al., 2014b). However, the repeats from 4 females deviated from these common building blocks and are more rare in the general population (Eichler et al., 1996; Yrigollen et al., 2014b). PCR-based assays might struggle to generate the correct repeat structure for these females as they use common haplotypes to infer the repeat structure of females whose X chromosomes camouflage each other's repeat structure (Chen et al., 2010). In another female a CTG interruption was detected within the CGG repeat which has not been reported so far. Most interruptions are AGG triplets, although also a TGG interruption was discovered by Kunst and Warren (1994) in a male sample. Possibly, also these alternative interruptions might stabilize the CGG repeat. Systematic mapping and collection on the transmission of repeats carrying those rare interruptions would provide insights in the stability of such repeats. It remains unfortunate that the PCR-based AGG assays cannot detect novel interruptions and hence impede further characterization of these unusual interruptions.

To conclude, AGG analysis by single-molecule sequencing generates clear results which contribute to determining an accurate expansion risk for females with a premutation and hence positively impacts genetic counseling. By incorporating AGG analysis into our diagnostic setting we also plan to investigate mother-daughter transmissions which can further help fine-tuning the risk estimates belonging to specific repeat classes.

## Author contributions

SA designed and performed all laboratory work and analyzed the data. HV, TdR, and KD ascertained the patients included in the study, interpreted the data and provided genetic counseling. VR and GM facilitated the diagnostic implementation. SA wrote the article which was critically revised and approved by VR, TdR, HV, KD, GM, and JV. JV coordinated the study and supervised the organization of the whole process.

### Conflict of interest statement

The authors declare that the research was conducted in the absence of any commercial or financial relationships that could be construed as a potential conflict of interest.
